# Whole Genome Sequencing of *Mycobacterium tuberculosis* Clinical Isolates From India Reveals Genetic Heterogeneity and Region-Specific Variations That Might Affect Drug Susceptibility

**DOI:** 10.3389/fmicb.2019.00309

**Published:** 2019-02-26

**Authors:** Jayshree Advani, Renu Verma, Oishi Chatterjee, Praveen Kumar Pachouri, Prashant Upadhyay, Rajesh Singh, Jitendra Yadav, Farah Naaz, Raju Ravikumar, Shashidhar Buggi, Mrutyunjay Suar, Umesh D. Gupta, Akhilesh Pandey, Devendra S. Chauhan, Srikanth Prasad Tripathy, Harsha Gowda, T. S. Keshava Prasad

**Affiliations:** ^1^Institute of Bioinformatics, International Technology Park, Bengaluru, India; ^2^Center for Systems Biology and Molecular Medicine, Yenepoya Research Centre, Yenepoya (Deemed to be University), Mangalore, India; ^3^Manipal Academy of Higher Education, Manipal, India; ^4^School of Biotechnology, Amrita Vishwa Vidyapeetham, Kollam, India; ^5^Department of Microbiology and Molecular Biology, ICMR-National JALMA Institute for Leprosy and Other Mycobacterial Diseases, Agra, India; ^6^Department of Neuromicrobiology, Neurobiology Research Centre, National Institute of Mental Health and Neurosciences, Bengaluru, India; ^7^Intermediate Reference Laboratory, State Tuberculosis Training and Demonstration Centre, Someshwaranagar, SDSTRC and RGICD Campus, Bengaluru, India; ^8^Department of Cardio Thoracic Surgery, Super Specialty State Referral Hospital for Chest Diseases, Someshwaranagar First Main Road, Dharmaram College Post, Bengaluru, India; ^9^School of Biotechnology, Kalinga Institute of Industrial Technology, Bhubaneswar, India; ^10^McKusick-Nathans Institute of Genetic Medicine, Johns Hopkins University School of Medicine, Baltimore, MD, United States; ^11^Department of Biological Chemistry, Johns Hopkins University School of Medicine, Baltimore, MD, United States; ^12^Department of Pathology, Johns Hopkins University School of Medicine, Baltimore, MD, United States; ^13^Department of Oncology, Johns Hopkins University School of Medicine, Baltimore, MD, United States

**Keywords:** fluoroquinolones, metagenomics, molecular genotyping, mycobacterial genetic heterogeneity, next generation sequencing

## Abstract

Whole genome sequencing (WGS) of *Mycobacterium tuberculosis* has been constructive in understanding its evolution, genetic diversity and the mechanisms involved in drug resistance. A large number of sequencing efforts from across the globe have revealed genetic diversity among clinical isolates and the genetic determinants for their resistance to anti-tubercular drugs. Considering the high TB burden in India, the availability of WGS studies is limited. Here we present, WGS results of 200 clinical isolates of *M. tuberculosis* from North India which are categorized as sensitive to first-line drugs, mono-resistant, multi-drug resistant and pre-extensively drug resistant isolates. WGS revealed that 20% of the isolates were co-infected with *M. tuberculosis* and non-tuberculous mycobacteria species. We identified 12,802 novel genetic variations in *M. tuberculosis* isolates including 343 novel SNVs in 38 genes which are known to be associated with drug resistance and are not currently used in the diagnostic kits for detection of drug resistant TB. We also identified *M. tuberculosis* lineage 3 to be predominant in the northern region of India. Additionally, several novel SNVs, which may potentially confer drug resistance were found to be enriched in the drug resistant isolates sampled. This study highlights the significance of employing WGS in diagnosis and for monitoring further development of MDR-TB strains.

## Introduction

Tuberculosis is one of the leading causes of death worldwide. The emerging drug resistance is a serious global threat and poses significant challenge to public health. According to the recent WHO report, there were an estimated 10.0 million cases of TB and 1.3 million deaths during the year 2017. India alone accounted for 24% of global MDR-TB incidence and 27% of global TB incidence among HIV-negative individuals (Global Tuberculosis Who Report [GWTR]x, 2018). There is an urgent need for improved diagnosis of TB, such as identification of markers to monitor transmission and effective treatment to deal with this deadly disease. Whole genome sequencing (WGS) studies from across the globe have revealed genetic diversity of *Mycobacterium tuberculosis* and have provided significant insights into its evolution and transmission (Casali et al., 2014; Walker et al., 2015; Nikolayevskyy et al., 2016). They have also revealed specific genotypes associated with drug resistance.

Several studies have shown association of the genetic variations with pathogenesis and drug resistance (Laurenzo and Mousa, 2011; Zhang et al., 2013; Casali et al., 2014). Global frontline molecular diagnostics such as line probe assays and Xpert MTB/RIF used for diagnosis of drug resistant TB, have been developed based on these genetic markers (Gagneux and Small, 2007; Thakur et al., 2015; Thirumurugan et al., 2015). However, these tests rely on a limited number of mutations. There have been several instances where phenotypic resistance could not be explained by known mutations associated with drug resistance (Rigouts et al., 2013; Banu et al., 2014; Ahmad et al., 2016). A recent study comparing the efficacy of Xpert MTB/RIF with line probe assay for detection of rifampicin mono-resistant *M. tuberculosis* reported the utility of country specific probes, to increase the sensitivity of Xpert MTB/RIF in India (Rufai et al., 2014). Since there is considerable genetic heterogeneity among *M. tuberculosis* isolates from different geographic regions, large-scale sequencing efforts are required to map genetic variations and identify the genotypes associated with drug resistance.

Whole genome sequencing studies for mapping genetic heterogeneity and identifying determinants of drug resistance among clinical isolates in India are limited (Chatterjee et al., 2017; Manson et al., 2017a). Previous studies have revealed that lineage 1 (Indo-Oceanic) and lineage 3 (East-African-Indian) are most prevalent in India and are less common in other parts of the world (Gutierrez et al., 2006; Ahmed et al., 2009). Lineage 1 is prevalent in South India whereas lineage 3 is prevalent in North India. A recent study has reported WGS data for 223 clinical isolates from South India (Manson et al., 2017a). They have observed genetic diversity among the sequenced isolates and reported potential novel genotypes that might be associated with drug resistance. The study has also observed potential mixed infections that might affect prediction of drug resistant phenotypes based on genotype data. In the current study, we performed WGS analysis of 200 culture confirmed *M. tuberculosis* clinical isolates from North India. We analyzed isolates from different categories such as sensitive to first line drugs, rifampicin mono-resistant, isoniazid mono-resistant, streptomycin mono-resistant, MDR and pre-extensively drug resistant (pre-XDR). We compared genetic variations observed in these isolates with data from 2,044 *M. tuberculosis* clinical isolates available in public domain. In addition, we also compared the genetic variations with those found in isolates prevalent in South India. We identified several novel genetic variations and novel genotypes in clinical isolates from North India that might potentially be associated with drug resistance.

## Materials and Methods

### Bacterial Isolates

Two hundred *M. tuberculosis* clinical isolates were collected from the Mycobacterial Repository Centre of the National JALMA Institute of Leprosy and Other Mycobacterial Diseases, Agra, India. This study was carried out in accordance with the recommendations of the guidelines of the National JALMA Institute of Leprosy and other Mycobacterial Diseases. The study was approved by the Institutional Ethics Committee of the National JALMA Institute of Leprosy and other Mycobacterial Diseases, Agra. All the subjects gave written informed consent in accordance with the Declaration of Helsinki.

### *M. tuberculosis* Culture and DNA Isolation for Whole Genome Sequencing

*Mycobacterium tuberculosis* clinical isolates used in the current study were cultured and maintained at the Department of Microbiology, the National JALMA Institute of Leprosy and other Mycobacterial Diseases, Agra, India. The isolates were procured from pulmonary sites of patients diagnosed with tuberculosis. The samples were processed as per the standard *N*-acetyl-L-cysteine-sodium hydroxide (NALC-NaOH) and inoculated on Lowenstein-Jensen (LJ) slants for primary culture (Apers et al., 2003; Steingart et al., 2006; Peres et al., 2009). The cultures were subsequently tested using biochemical assays to rule out NTM or any other infection. The samples were sub cultured using single colony growth on Lowenstein-Jensen (LJ) for DNA isolation. DNA libraries were constructed with genomic DNA extracted using CTAB method (van Soolingen et al., 1991). DNA was fragmented in the range of 100 to 800 bases. The fragmented DNA was cleaned up using QIAquick columns (QIAGEN). The size distribution was checked by running aliquots of the samples on Agilent Bioanalyzer 7500 Nano chips. Illumina adapters were ligated to each fragment. Fragments of ∼300 bases were separated using Gel electrophoresis and sequenced at both ends using Illumina HiSeq 2500 sequencer. Sequencing depth in all the isolates was more than 100X with average 100 bp paired end read length.

### Drug Susceptibility Testing

Drug susceptibility testing of *M. tuberculosis* isolates was performed using minimal inhibitory concentration (MIC) method on LJ slants. Drug concentrations used for testing were- 64 μg/ml for Streptomycin sulfate (STR), 1 μg/ml for Isoniazid (INH), 64 μg/ml for Rifampicin (RMP), 6 μg/ml for Ethambutol (EMB), 64 μg/ml for Kanamycin (KAN), and 6 μg/ml for Ofloxacin (OFX) (Canetti et al., 1969; Singh et al., 2009; Purwar et al., 2011). All drugs were procured from Sigma Aldrich. Inoculated LJ slants were examined for growth after 28 days of incubation and the colony count was recorded. The strain was considered resistant if the colony count on the slant was 20 or more (Canetti et al., 1969). Isolates were classified into the following categories during data analysis on the basis of drug resistance profiles: Sensitive to first line drugs (*n* = 50), MDR (*n* = 91) Rifampicin mono-resistant (*n* = 12), Isoniazid mono-resistant (*n* = 31), Streptomycin mono-resistant (*n* = 6), Pre-XDR (*n* = 10).

### Mapping and Variant Detection Using H37Rv Reference Genome

The quality of the raw reads was checked using FastQC version-0.11.5 toolkit followed by trimming of adapters and low-quality bases with a Phred quality score of less than 20. Reads were then mapped on to H37Rv reference genome (NC_000962.3) using Burrows-Wheeler Alignment Tool (BWA) (version-0.7.15) (Li and Durbin, 2009). The alignment files were subjected to local realignment and de-duplication using the Genome Analysis Toolkit (GATK) (version-3.6) and Picard. Variants SNVs and Insertion/Deletions (In/Dels) were called from each alignment file using GATK and Pindel (version 0.2.5b8) (Ye et al., 2009). Variant filtering was carried out by removing variants with <5 reads. Variants with low base quality of <20 were discarded. The variants were annotated using the scripts^1^. Variants selected had a minimum read depth of 5 reads and a mapping quality score of above 20. All variants identified in this study were manually inspected using Integrative Genomics Viewer (IGV) (version-2.3.86) (Ye et al., 2009).

### Metagenomics Analysis

Metagenomic analysis was performed using Kraken which, is a program for assigning taxonomic labels to short DNA sequences obtained through metagenomic studies (Wood and Salzberg, 2014). This analysis was carried out for 39 isolates where we observed poor sequence alignment with *M. tuberculosis* H37Rv genome.

### Principal Component Analysis

Sequence variants from GMTV and NIRT datasets were used along with our dataset to carry out principal component analysis (PCA) (Chernyaeva et al., 2014; Manson et al., 2017b). The Genome-wide *Mycobacterium tuberculosis* Variation (GMTV) database is a cumulative catalog of all genetic variations observed in *M. tuberculosis* strains from all over world and the NIRT data comprises of genome information on 220 *M. tuberculosis* isolates sequenced recently from South India (Chernyaeva et al., 2014; Manson et al., 2017b). A total of 87,613 SNVs from 2,201 isolates was used to generate the matrix of SNVs. PCA was performed using ggfortify package in R version-3.2.0.

### Phylogenetic Analysis and Spoligotyping

*Mycobacterium tuberculosis* complex lineages/sublineages and *in silico* spoligotyping were determined using KvarQ (version-0.12.2) (Steiner et al., 2014). SNVs identified in each isolate were used to construct the phylogenetic tree. Individual consensus sequence fasta files for isolates with SNVs identified in each of the isolates were generated using vcf-consensus module (Applies VCF variants to a fasta file to create consensus sequence) from VCFtools (Version 0.1.16) (Danecek et al., 2011). Multiple sequence alignment was carried out using MAFFT tool for the consensus sequences fasta files for all the isolates (Version 7.407) (Nakamura et al., 2018). The output file generated after multiple sequence alignment using MAFFT was further used to constructing the phylogenetic tree. The maximum-likelihood method was implemented in IQ-TREE for constructing the phylogenetic tree (Nguyen et al., 2015). The output was visualized with Dendroscope (version-3.5.8) (Huson and Scornavacca, 2012).

### Identification of Drug Resistance-Associated Genes and SNVs

We compiled list of genes associated with drug resistance from literature and databases and a list of non-synonymous SNVs observed in these genes among drug-resistant isolates (Supplementary Table S5).

Different drug resistant categories (Sensitive to first line drugs, MDR, Rifampicin mono-resistant, Isoniazid mono-resistant, Streptomycin mono-resistant, Pre-XDR) were used to identify the SNVs that were observed in these genes among drug-resistant isolates. The proportion of drug-resistant isolates (FR) in which a given gene was mutated minus the proportion of drug-sensitive isolates (FS) in which the same gene was mutated (i.e., FR – FS) was calculated, The mean value and standard deviation of this dataset was used to obtain the quantile *P*-value (*P* < 0.05) of the corresponding normal distribution for each gene (Zhang et al., 2013). The resulting non-synonymous SNVs were designated as relatively high-frequency non-synonymous SNVs in the drug-resistant isolates. A two-tailed *t*-test was used to compare and characterize the significant mutations. Bonferroni-corrected *P-*values were calculated using the hyper geometric function (entries with *P*-value ≤ 0.05).

### Co-occurring and Compensatory Mutations in Drug Resistant Isolates

The matrix of mutations associated with drug resistance for isolates from our dataset and isolates from NIRT study was generated to identify co-occurring and compensatory mutations (Manson et al., 2017b).

## Results

We carried out WGS of 200 culture confirmed *M. tuberculosis* clinical isolates from North India spanning a broad range of drug resistance profiles (50 sensitive to four first line drugs, 12 rifampicin mono-resistant, 31 isoniazid mono-resistant, 6 streptomycin mono-resistant, 91 MDR and 10 Pre-XDR) (Supplementary Table S1). The isolates were collected over a period of 4 years (2010–2014) at the National JALMA Institute of Leprosy and other Mycobacterial Diseases, Agra, India. We compared genetic diversity between clinical isolates from North India and South India. In addition, we determined novel genetic variations observed in isolates from India when compared with publicly available WGS data of isolates from rest of the world.

### Whole Genome Sequencing Data Reveals Co-infections Undetected in Culture-Based Diagnosis

**FIGURE 1:**
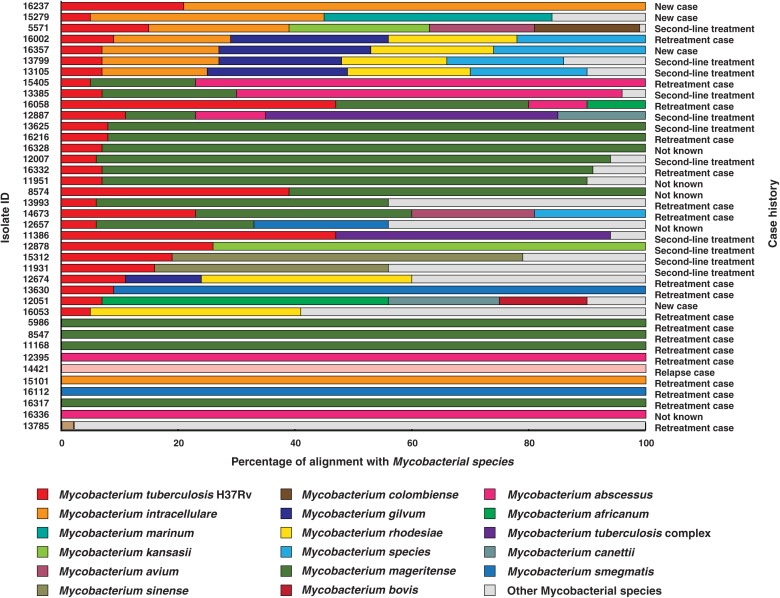
Isolates identified as co-infections. The length of bars indicates proportion of reads that were aligned to corresponding mycobacterial species. (WHO definitions for case history. (i) New case- Patient has never been treated for TB. (ii) Retreatment case- Patient on retreatment regimen with first-line drugs. (iii) Second-line treatment- Patient has been started directly on second-line treatment for MDR-TB or RR-TB, without being started on a first line drugs. (iv) Relapse case- Patient has been previously treated for TB and are now diagnosed with a recurrent episode of TB. (v) Not known- Case history unavailable).

The *M. tuberculosis* genome is 4.4 million base pairs long and encodes for approximately 4,000 genes (Cole et al., 1998). All the 200 culture confirmed isolates sequenced in this study were aligned against *M. tuberculosis* H37Rv reference genome (NC_000962.3). We observed poor sequence alignment for 39 isolates (Supplementary Table S1). As co-infections are generally suspected in TB, we performed alignment of these 39 isolates with multiple non-tuberculous mycobacterium species. Our analysis indicated co-infection (Figure 1).

The reads from 20% (*n* = 39) of the clinical isolates in our study aligned to genomes of multiple mycobacterial species, of which 9 were found to be NTMs and the other 30 were co-infection cases with *M. tuberculosis*. We observed *M. mageritense* to be prevalent in 46% (14/30) of the cases followed by *M. intracellulare* (7/30) and *M. abscessus* (4/30). *M. mageritense* is a recently described, non-pigmented infectious mycobacterium with closest similarity to the *M. fortuitum* third biovariant complex (Domenech et al., 1997). These clinical isolates were earlier reported to contain only *M. tuberculosis* by conventional culture-based methods. Of the 30 co-infection with *M. tuberculosis* cases 36% (11/30) were tuberculosis relapse cases and 53% (16/30) were resistant to first line anti-tubercular drugs (MDR) as per phenotypic DST (Figure 1). Our findings are in line with previous reports that have observed high prevalence of co-infections in cases with multidrug resistance and which results in adverse treatment outcomes (Gopinath and Singh, 2009; Fusco da Costa et al., 2013).

### Genome-Wide Sequence Variations in *Mycobacterium tuberculosis*

**FIGURE 2:**
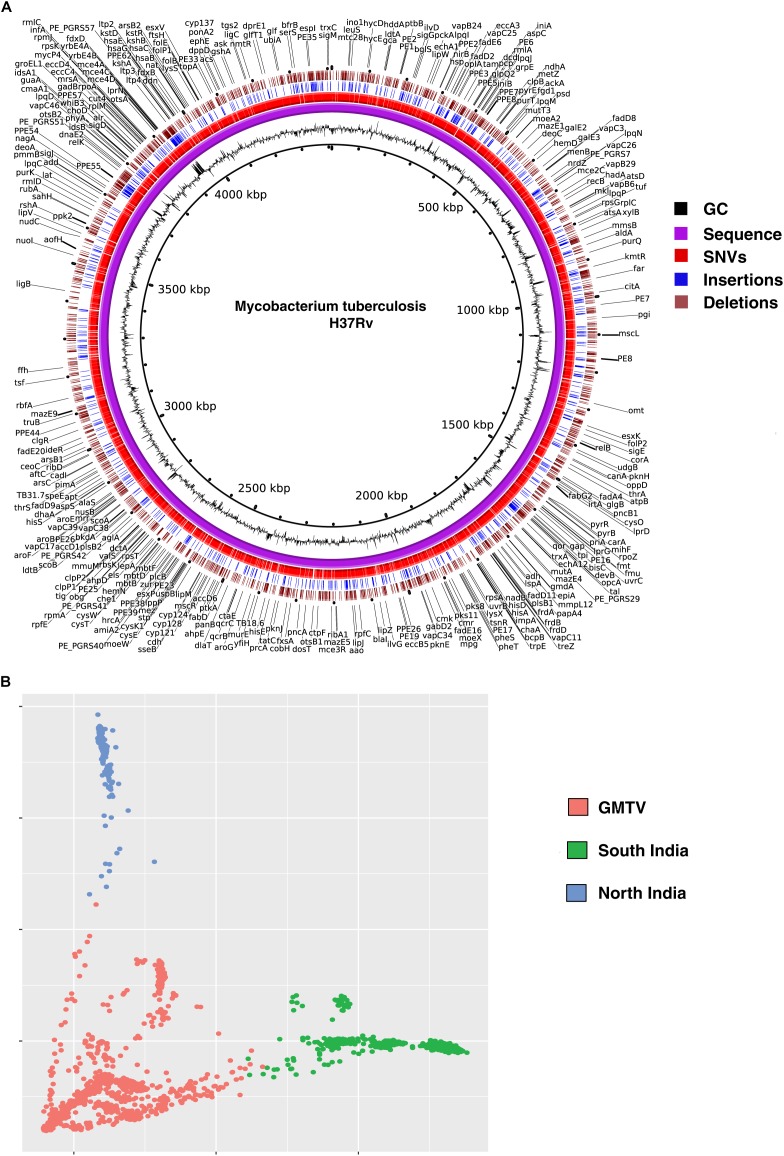
Genetic variations in *Mycobacterium tuberculosis* isolates. **(A)** Circos plot depicting novel SNVs and INDELS. **(B)** Principal Component Analysis (PCA) of non-synonymous SNVs from GMTV, isolates from South India and isolates from the current study (North India). Blue, green, and pink dots represent isolates from North India, South India and GMTV database, respectively.

We called SNVs, insertions and deletions from the remaining 161 *M. tuberculosis* clinical isolates. A total of 18,970 non-synonymous SNVs (Supplementary Table S2), 3,052 insertions (Supplementary Table S3) and 2,739 deletions (Supplementary Table S4) were identified when compared with H37Rv genome. We compared the SNVs identified in our dataset with (i) those reported in genome-wide *M. tuberculosis* variation database (GMTV) (Chernyaeva et al., 2014), which has data from 2,044 *M. tuberculosis* isolates around the globe and (ii) 223 isolates from South India (Manson et al., 2017b). There were 12,802 SNVs that were unique to our dataset when compared to these publicly available datasets (Figure 2A). We carried out PCA to determine genetic diversity of isolates based on sequence variations. We used sequence variations cataloged from global data in GMTV, our dataset from North India and dataset from South India. PCA revealed distinct clustering of isolates from GMTV, North India and South India indicating genetic diversity of isolates from different regions (Figure 2B).

### Phylogenetic Analysis of *M. tuberculosis* Isolates

**FIGURE 3:**
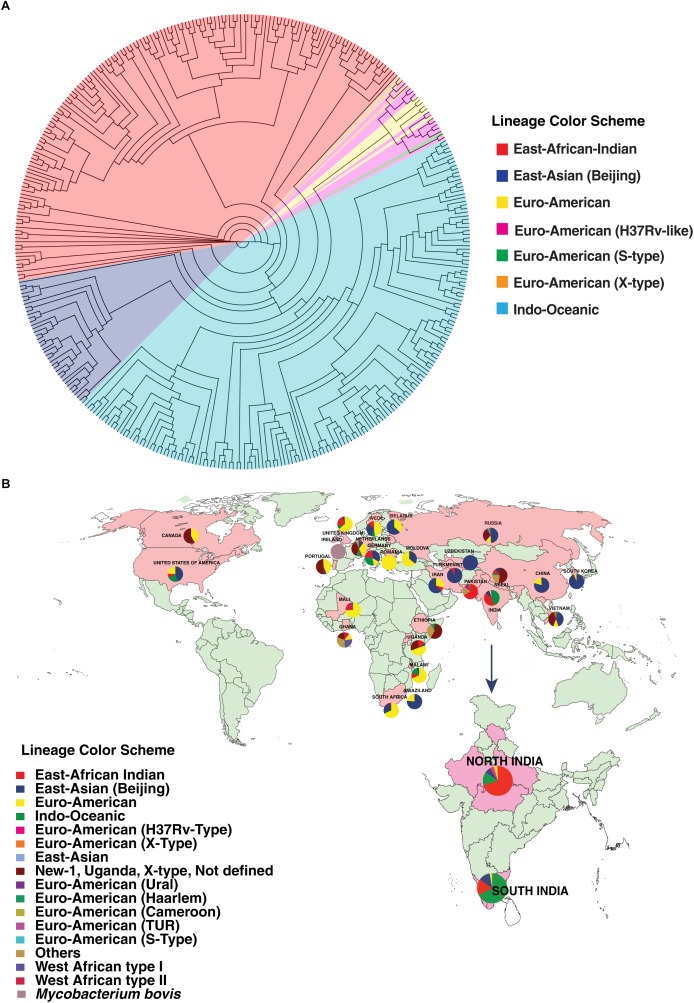
Distribution of isolates. **(A)** Phylogenetic clustering of *M. tuberculosis* isolates from North and South India. **(B)** Geographical distribution of different *M. tuberculosis* lineages across the globe.

We carried out phylogenetic analysis of 161 confirmed cases of *M. tuberculosis* isolates to determine lineage distribution. Phylogenetic analysis revealed that the isolates in our study belonged to seven different lineages with East-African-Indian (Lineage 3, *n* = 114) being the most common. Other lineages include Indo-Oceanic (Lineage 1, *n* = 23), East-Asian (Beijing) (Lineage 2.2, *n* = 10), Euro-American (Lineage 4, *n* = 6), Euro-American (X-type) (Lineage 4.1.1, *n* = 1) and Euro-American (H37Rv-like) (Lineage 4.9, *n* = 7) (Table 1). To examine Indian phylogeographical patterns, phylogenetic clustering of isolates from our study (North India) and isolates from South India was performed (Manson et al., 2017b). While East-African-Indian (lineage 3) was found prevalent in North India (70%, 114/161), Indo-Oceanic lineage was most prevalent in South (67.7%, 151/223) (Figure 3A). This observation is in agreement with previous findings (Gutierrez et al., 2006; Ahmed et al., 2009). East-African-Indian and Indo-Oceanic lineages are most prevalent in Indian subcontinent compared to elsewhere (Figure 3B).

**Table 1:** Overall distribution of *M. tuberculosis* clinical isolates on the basis of lineage.

Profile	Indo-Oceanic (Lineage 1)	East-Asian (Beijing) (Lineage 2)	East-African-Indian (Lineage 3)	Euro-American (Lineage 4)	Euro-American (X-type) (Lineage 4.1.1)	Euro-American (H37Rv-like) (Lineage 4.9)
Sensitive to first line drugs	4 (2.4%)	0 (0%)	28 (17.4%)	2 (1.2%)	1 (0.6%)	2 (1.2%)
MDR	14 (8.6%)	6 (3.7%)	46 (28.6%)	2 (1.2%)	0 (0%)	3 (1.8%)
Isoniazid mono-resistant	3 (1.8%)	0 (0%)	23 (14.2%)	1 (0.6%)	0 (0%)	2 (1.2%)
Rifampicin mono-resistant	1 (0.6%)	0 (0%)	10 (6.2%)	0 (0%)	0 (0%)	0 (0%)
Streptomycin mono-resistant	0 (0%)	0 (0%)	5 (3.1%)	0 (0%)	0 (0%)	0 (0%)
Pre-XDR	1 (0.6%)	4 (2.4%)	2 (1.2%)	1 (0.6%)	0 (0%)	0 (0%)


### Mutations Associated With Drug Resistance

**FIGURE 4:**
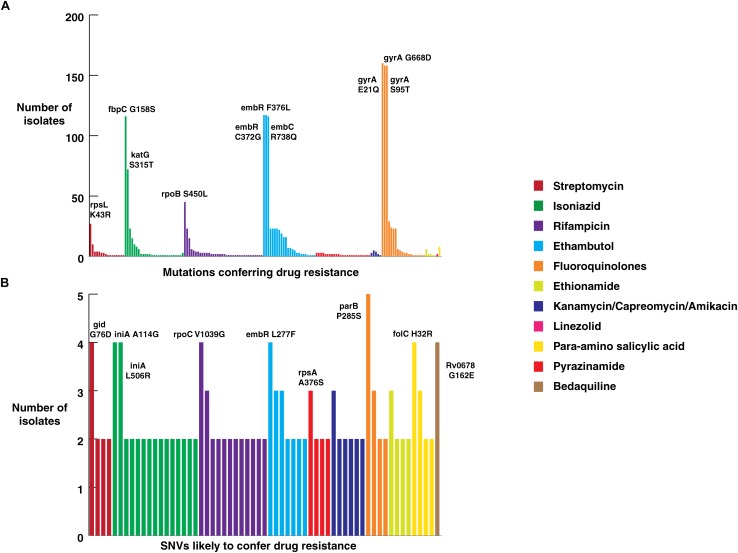
Frequency of SNVs that confer drug resistance. **(A)** Frequency of SNVs known to confer resistance against first and second-line anti-tubercular drugs in clinical isolates from North India. **(B)** Frequency of novel SNVs in genes that confer drug resistance.

All isolates sequenced in this study were tested for phenotypic resistance for four first line drugs (rifampicin, isoniazid, ethambutol, and streptomycin). In addition, 10 of them were also tested for two second-line drugs kanamycin and ofloxacin (Supplementary Table S1). We compared our SNV data with known drug resistance causing SNVs. A total of 160 previously reported SNVs in 25 genes that are known to confer resistance to first- and second-line drugs were identified in our dataset (Supplementary Table S5) (Figure 4A). Of the 160 known SNVs, 35% (56/161) of the SNVs were exclusive to MDR-TB clinical isolates. In addition, 343 novel SNVs were identified in 38 genes, SNVs in which have been previously reported to confer drug resistance (Supplementary Table S6) (Figure 4B). We also observed 1,553 non-synonymous SNVs corresponding to 974 genes that were significantly enriched in drug resistant isolates compared to sensitive isolates (*P*-value ≤ 0.05) (Supplementary Table S7). Thus, we reasoned that drug resistant isolates should potentially be enriched with SNVs conferring resistance first and second-line drugs.

Mutations in 81-bp rifampicin resistance-determining region (RRDR) of *rpoB* gene, also known as hot spot region, have been accurate predictors of rifampicin resistance (Telenti et al., 1993). We observed 21 known SNVs within this region in our dataset. The most common rifampicin resistance genotype, encoding a (S450L) substitution was identified in 28% (45/161) of clinical isolates (Mandin et al., 2007). We found the *katG* (S315T) SNV that confers resistance to isoniazid in 45% (72/161) of the isolates. Of the 72 isolates that carried this *katG* (S315T) SNV, 47 were MDR as per first line DST. On the other hand, SNV (S450L) in *rpoB* gene, which confers resistance to rifampicin, was found only in 28% (45/161) of the isolates.

### High Prevalence of Fluoroquinolone Resistant Genotypes in *M. tuberculosis*

**FIGURE 5:**
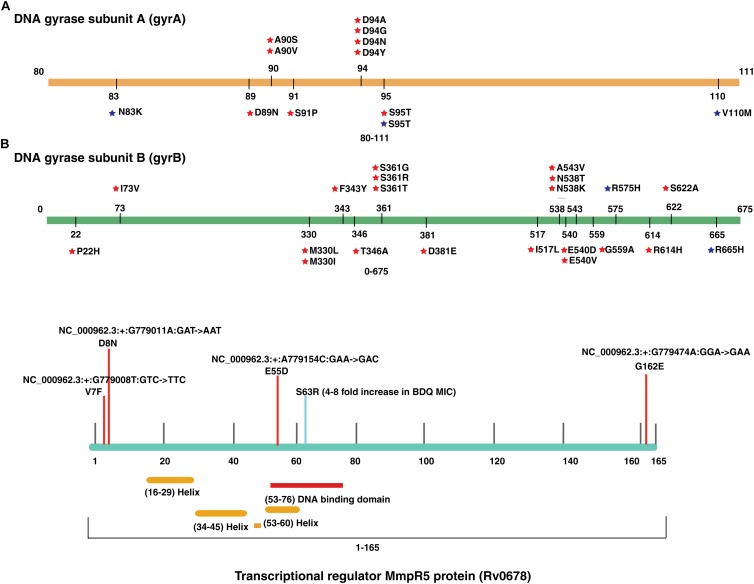
Single nucleotide variations in genes that confer resistance to fluoroquinolone and bedaquiline. **(A)** SNVs in quinolone resistance-determining regions (QRDRs) of *gyrA* and *gyrB* genes. SNVs marked in blue are reported in isolates obtained from South India and SNVs marked in red are the ones identified in our study. **(B)** SNVs in *Rv0678* gene that potentially confer bedaquiline resistance. SNVs marked in blue are reported earlier and SNVs marked in red are novel.

We evaluated the prevalence of fluoroquinolone resistance among isolates sequenced in our study by looking at *gyrA* and *gyrB* gene mutation frequencies. We identified 26 SNVs in *gyrA* gene of which 10 are known to confer drug resistance to fluoroquinolones. Of the 10 known SNVs in *gyrA* gene, 8 were found to affect quinolone resistance-determining regions (QRDR) region. Additionally, the known SNV (S95T) in *gyrA* gene was found in 158 isolates. The *gyrB* gene was found to harbor 19 SNVs outside the QRDR region. Eight of these SNVs were found in 20% (33/161) isolates that are known to confer drug resistance. The SNV in (D94N) in *gyrA* gene which is known to confer fluoroquinolone resistance was found to be more prevalent in pre-XDR isolates. This SNV was identified in six isolates of which four were pre-XDR. The isolates sampled in the dataset from South India also display a similar frequency of SNV in *gyrA* gene thus, indicating the high prevalence of fluoroquinolone resistance in India (Figure 5A and Table 2).

**Table 2:** List of SNVs known to confer resistance to fluoroquinolones.

(A) Distribution of fluoroquinolones resistance in isolates from North India									

Gene symbol	Mutation	Total	Sensitive to first line drugs	MDR	Isoniazid mono-resistant	Rifampicin mono-resistant	Streptomycin mono-resistant	Pre-XDR	*P-*value
*gyrB*	M330I	23	4	15	1	1	–	1	2.13E-03
*gyrA*	E21Q	160	36	71	29	11	5	8	3.68E-28
*gyrA*	A90V	24	1	20	1	1	–	1	1.10E-02
*gyrA*	D94N	6	–	1	1	–	–	4	3.67E-02
*gyrA*	D94G	29	1	24	2	1	–	1	4.75E-03
*gyrA*	S95T	158	35	70	29	11	5	8	5.32E-28
*gyrA*	A384V	23	4	14	3	1	–	1	5.02E-04
*gyrA*	G668D	158	35	70	29	11	5	8	5.32E-28

**(B) Distribution of fluoroquinolones resistance in isolates from South India**									

**Gene symbol**	**Mutation**	**Total**	**Susceptible**	**MDR**	**monoDR**	**polyDR**			***P-*value**

*gyrB*	M330I	–	–	–	–	–	–	–	–
*gyrA*	E21Q	220	168	16	29	7	–	–	6.319E-22
*gyrA*	A90V	–	–	–	–	–	–	–	–
*gyrA*	D94N	–	–	–	–	–	–	–	–
*gyrA*	D94G	–	–	–	–	–	–	–	–
*gyrA*	S95T	219	167	16	29	7	–	–	6.714E-22
*gyrA*	A384V	149	117	5	21	6	–	–	2.215E-14
*gyrA*	G668D	219	167	16	29	7	–	–	6.714E-22

**(C) Distribution of fluoroquinolones resistance in isolates listed in GMTV database**									

**Gene symbol**	**Mutation**	**Total**	**Susceptible**	**MDR**	**Isoniazid mono-resistant**	**Rifampicin mono-resistant**	**Streptomycin mono-resistant**	**Pre-XDR**	***P-*value**

*gyrB*	M330I	–	–	–	–	–	–	–	–
*gyrA*	E21Q	814	293	400	28	7	20	66	7.434E-91
*gyrA*	A90V	29	3	17	1	–	–	8	7.655E-06
*gyrA*	D94N	17	3	7	–	–	–	7	2.336E-08
*gyrA*	D94G	67	5	43	–	–	–	19	5.402E-14
*gyrA*	S95T	739	246	383	22	4	18	66	6.139E-80
*gyrA*	A384V	–	–	–	–	–	–	–	–
*gyrA*	G668D	738	246	382	22	4	18	66	6.259E-80


### Emergence of Bedaquiline Resistance in India

We identified a novel non-synonymous SNV (G162E) in *Rv0678* gene in three MDR and one isoniazid mono-resistant clinical isolate in our dataset (Figure 5B). In addition, we also observed SNV (N148H) in three isolates from South India. The *Rv0678* gene is known to be associated with bedaquiline resistance. Keeping in mind that bedaquiline is a fairly new anti-TB drug; the identification of SNVs in this gene suggests the dynamic and rapid evolution of *M. tuberculosis* clinical strains.

### Co-occurring and Compensatory Mutations in Drug Resistant Isolates

**FIGURE 6:**
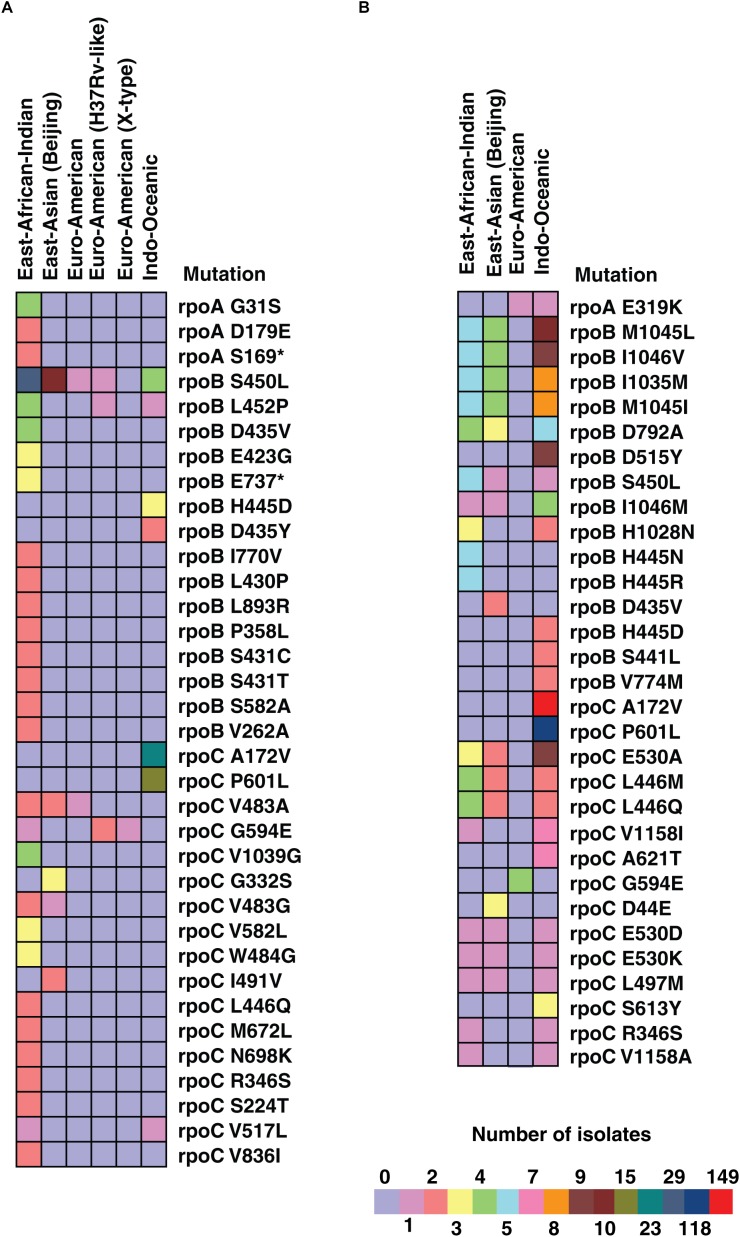
Number of SNVs in *rpo A, B*, and *C* genes in different lineages in isolates from North and South India. **(A)** Colored bands represent number of SNVs in *rpo A, B*, and *C* genes in isolates from North India across different lineages. **(B)** Colored bands represent number of SNVs in *rpo A, B*, and *C* genes in isolates from South India across different lineages.

We investigated the occurrence of compensatory mutations in *rpoC* gene in the isolates carrying rifampicin resistance mutations in *rpoB* gene. Of the 51 isolates carrying SNVs in *rpoB* gene, 20% (10/51, *P-*value ≤ 0.05) were found to carry a SNV in *rpoC* gene (Figure 6). We observed (S450L) SNV in *rpoB* gene among 3.4% of isolates from South India compared to 25% among isolates from North India. However, the frequency of compensatory mutation (A172V) in *rpoC* gene in the same isolates was overwhelmingly high in isolates from South India (74%) than in North India (12.5%) (Supplementary Figure S1). In addition to identification of mutations in *rpo* genes, we also identified four major clusters of co-occurring mutations highly prevalent in specific lineages. The SNV (D229G) in *accD6* gene (Rv2247) was exclusively found to be co-occurring with *katG* (R463L) in all East-Asian (Beijing) clade MDR and Pre-XDR isolates. Similarly, the *gid* (E92D) SNV was found to co-occur with *rpsL* (K43R) and was prevalent in East-Asian (Beijing) clade with MDR and pre-XDR drug resistance phenotype. We also observed a cluster of SNVs associated with ethambutol resistance, which were overrepresented with higher prevalence in East-African-Indian MDR clinical isolates which include – 2 novel SNVs *embB* (G406A) and *embB* (M306I) and one known SNV *embB* (M306V) (*P-*value ≤ 0.05).

## Discussion

In this study, we carried out WGS of 200 culture confirmed *Mycobacterium tuberculosis* clinical isolates from a tertiary care centre in North India. WGS revealed 39 cases of *M. tuberculosis* co-infection with various species of NTM. Surprisingly, we observed *M. mageritense* to be the most prevalent NTM co-infection with *M. tuberculosis* unlike previous studies that have reported *M. avium* complex (MAC) and *M. abscessus*. NTM infections in high endemic countries such as India either go unrecognized or are misdiagnosed as pulmonary TB due to their indistinguishable clinical presentations (Gopinath and Singh, 2010). Several reports of concomitant identification of multiple infections with *M. tuberculosis*, *M. avium* and others have been published (Kox et al., 1995; Montessori et al., 1996; Fusco da Costa et al., 2013). Co-infections may lead to misidentification of strains and result in underestimation of disease transmission (Theisen et al., 1995; Niemann et al., 2000; van Rie et al., 2005). They also lead to erroneous drug-susceptibility profiles (DST) resulting in poor treatment outcomes. Using multiplex PCR assay, Gopinath and Singh (2009) have reported NTM infection in more than 17% of confirmed pulmonary TB cases suggesting a higher proportion of co-infections with *M. tuberculosis* cases missed during routine TB diagnosis. In addition, studies have also shown that majority of cases with co-infections were found to be drug resistant (Fusco da Costa et al., 2013).

Among several species of NTMs infecting humans, *M. avium* complex (MAC) and *M. abscessus* are reported to be more common (Gopinath and Singh, 2009; Nishiuchi et al., 2017). Most NTMs have been found to be either resistant or partially resistant to standard anti-tubercular drugs and require distinct treatment strategies (Gopinath and Singh, 2010). For example, *M. mageritense* is susceptible to ciprofloxacin and sulfamethoxazole and resistant to clarithromycin (Wallace et al., 2002). Further investigations are needed to evaluate if combined treatment would result in better outcomes among co-infected patients. Accurate diagnosis of these infections thus becomes critical for effective treatment. This highlights the significance of involving WGS approaches for diagnosis and treatment of tuberculosis.

Our study identified several novel genetic variations that were not previously reported in *M. tuberculosis*. Among these were several novel genetic variations in genes that are known to confer drug resistance. However, the implication of these novel SNVs on drug susceptibility is yet to be characterized. We also observed significant differences in frequency of these SNVs across different lineages. In a recent analysis of *M. tuberculosis* genomes from different countries, Manson et al. (2017b) have reported that majority of MDR-TB and XDR-TB strains evolved resistance to isoniazid before resistance to rifampicin. This is attributed to introduction of isoniazid for clinical use approximately 20 years before rifampicin resulting in selection of *M. tuberculosis* populations carrying mutations conferring isoniazid resistance. We observed similar trend from genotypic data on *katG* and *rpoB* in our dataset. Mutations in *rpoB* gene detected by Xpert MTB/RIF are those which occur later during infection. As other high-frequency mutations precede emergence of *rpoB* mutations, probes that monitor these high frequency mutations might be valuable to identify patients with higher risk of acquiring MDR-TB. The 343 novel SNVs identified in this study can further have important implications for designing rapid methods for detecting drug resistant strains. These findings signify that molecular assay kit diagnosis that is based on the *rpoB* and *katG* genes should be improved by incorporating SNVs associated with other drugs and considering geographic specificity.

TB strains that are resistant to any fluoroquinolone in addition to first line drugs and one of the three injectable second-line drugs, amikacin, kanamycin, or capreomycin are classified as extremely drug resistant (XDR) (Von Groll et al., 2009). Fluoroquinolones are a class of antibiotics, which inhibit DNA gyrases and thus prevent bacterial DNA synthesis. They play an important role in MDR-TB treatment (Moadebi et al., 2007). In addition to TB, fluoroquinolones have been extensively used for treatment of several bacterial infections including *S. pneumoniae* (Low, 2009). Increasing fluoroquinolone prescriptions and the expanded use of these broad-spectrum agents for many infections has resulted in emergence of fluoroquinolone resistance. Global surveillance studies demonstrate that fluoroquinolone resistance rates increased in the past years in almost all bacterial species (Dalhoff, 2012). This may be due to easy availability of these drugs without prescription in many places and indiscriminate use of them for undiagnosed respiratory tract infections (Dalhoff, 2012). Frequent cases of fluoroquinolone resistance in TB patients are impeding the use of this class of drug for TB treatment (Devasia et al., 2009). However, resistance to fluoroquinolones is not routinely evaluated in tuberculosis. Resistance to fluoroquinolones in *M. tuberculosis* is primarily due SNVs in *gyrA* or *gyrB* genes affecting the quinolone resistance-determining regions (QRDRs) that interacts with the drugs (Devasia et al., 2012; Maruri et al., 2012; Li et al., 2014). This data strongly suggests that more studies should be carried out from India which would contribute to the development, refinement, and improvement of rapid molecular diagnostic tests for determining fluoroquinolone resistance. This will also help in identification of MDR and pre-XDR TB in more timely and effective manner.

Bedaquiline is a fairly recent anti-tuberculosis drug. It has shown high potency against multi and extremely drug resistant cases. As of 2017, approximately 12,194 patients have been treated with bedaquiline (Tiberi et al., 2018). It was recently granted accelerated approval by FDA as part of combination therapy to treat adults with MDR-TB when an effective treatment regimen cannot otherwise be provided (Zumla et al., 2014). It targets ATP synthase activity in *M. tuberculosis* (Andries et al., 2005). Unfortunately, the rate at which mycobacteria are acquiring resistance to this newly introduced drug across the globe is alarming (Andries et al., 2014; Nguyen et al., 2017). A recent study showed that there is an alarming rate of increase in bedaquiline resistance in both patients with or without previous TB-treatment exposure (Veziris et al., 2017). It has been previously described that mutations in *atpE gene* (ATP synthase subunit c) is associated with bedaquiline resistance (Andries et al., 2014). However, target-based assays showed that approximately 30% of the isolates which are resistant to bedaquiline carry mutations in ATP synthase genes suggesting an alternate mechanism of resistance that does not involve drug target (Huitric et al., 2010; Ismail et al., 2018). Andries colleagues have shown that mutations in *Rv0678* gene, which is not a target of bedaquiline, may account for non-target based resistance to bedaquiline and cross-resistance between clofazimine and bedaquiline. They demonstrated that *Rv0678*, which is also known as “mycobacterial membrane protein repressor” (MmpR5), is a negative regulator of MmpS5 and MmpL5. Mutations in this gene leads to an increased expression of this efflux pump. Several non-*atpE* mutants resistant to bedaquiline have been known to carry mutation in *Rv0678*, including missense mutations (G281A), single nucleotide insertions (Ins A 38–39), and IS*6110* insertion sequences (IS6110 nt 272) (Milano et al., 2009; Ioerger et al., 2013). Considering mutations that potentially confer resistance to bedaquiline already exist in some of the isolates, it is important to monitor the efficacy of bedaquiline and its resistance pattern.

Recent studies have suggested that the fitness cost of antimicrobial resistance can be mitigated by compensatory mutations (Andersson and Hughes, 2010; Borrell and Gagneux, 2011). For example, *M. tuberculosis* carrying mutation *rpoB* (S450L) that acquires a compensatory mutation in *rpoC* restores its fitness and allows bacteria to thrive without any notable reduction in growth (Zhang et al., 2013). Compensatory mutations associated with *rpoB* gene harboring mutations in the 81-bp rifampicin resistance-determining region have been well-studied. Mutations in some genes also tend to co-occur in drug resistant bacteria. We observed co-occurring SNVs in *gid* (E92D) and *rpsL* (K43R) genes among the East Asian (Beijing) clade isolates sequenced in our study. *gid* (E92D) is a lineage specific polymorphism associated with Beijing clade (Spies et al., 2011) whereas *rpsL* (K43R) is the most common *rpsL* mutation significantly associated with Beijing strains compared to non-Beijing strains (Sun et al., 2010). In addition, *gidB* is reported to be a highly polymorphic gene suggesting it as a proposed target of selective pressure (Wong et al., 2011). These co-occurring and compensatory mutations can further be validated on large number of isolates which can provide information on drug resistance and further can be used as a novel approach for optimizing current treatment regimens.

As novel SNVs may have direct implications on drug resistance; country specific probes would be needed for rapid and effective diagnosis and treatment of drug resistant TB. Identification of large number of genetic variations in *M. tuberculosis* isolates sequenced in our study also suggests that such large-scale efforts are needed for comprehensive characterization of genetic diversity of *M. tuberculosis*. This is particularly true for isolates from Indian subcontinent as they are relatively underrepresented in global datasets.

## Data Availability

The raw WGS data has been deposited in the NCBI Sequence Read Archive (SRA) (http://www.ncbi.nlm.nih.gov/sra) and can be accessed through the accession PRJNA379070.

## Author Contributions

TP, ST, and HG designed the study and managed the study. JA, RV, PP, JY, and RS collected isolates. RV, JA, PP, JY, RS, and FN cultured the isolates. PP, PU, RS, and JY performed drug susceptibility testing. RV, JA, and FN prepared DNA samples. JA performed whole genome sequencing analysis. RV and JA interpreted the data. JA, RV, and OC analyzed the data. RV, JA, OC, HG, and TP wrote the manuscript. OC, JA, and RV illustrated figures. TP, HG, ST, DC, AP, RR, SB, MS, and UG reviewed the manuscript. HG and TP directed the work. TP, HG, ST, AP, and DC designed and provided overall direction for the project.

## Conflict of Interest Statement

The authors declare that the research was conducted in the absence of any commercial or financial relationships that could be construed as a potential conflict of interest. The reviewer AK declared a shared affiliation, with no collaboration, with one of the authors, AP, to the handling Editor at the time of review.

## Footnotes

1 https://github.com/JayshreeAdvani/categoriseSNVs

## Supplementary Material

FIGURE S1 Co-occurring mutations in *M. tuberculosis* clinical isolates. The mutations marked in red are the co-occurring mutations. **(A)** The highlighted clusters show the prevalence of these mutations in different lineages of *M. tuberculosis* in isolates from North India. **(B)** The highlighted clusters show the prevalence of these mutations in different lineages of *M. tuberculosis* in isolates from South India.

TABLE S1 Patient details, characterization of isolates as per WGS analysis and drug susceptibility testing status of *M. tuberculosis* clinical isolates.

TABLE S2 List of SNVs identified in the study.

TABLE S3 **(A)** List of insertions identified in our study in the CDS region. **(B)** List of insertions identified in our study in the intergenic region.

TABLE S4 **(A)** List of deletions identified in our study in the CDS region. **(B)** List of deletions identified in our study in the intergenic region.

TABLE S5 List of SNVs in genes known to confer drug resistance and their relative frequency.

TABLE S6 List of novel SNVs associated with first- and second-line drug resistance identified in the study.

TABLE S7 List of SNVs that are identified in a higher proportion of drug resistant isolates than drug-sensitive isolates.

## Abbreviations

MDR: multi-drug resistant
MTB: *Mycobacterium tuberculosis*
MTBC: *Mycobacterium tuberculosis* complex
NCBI: National Centre for Biotechnology Information
NTM: non-tuberculous mycobacteria
SNVs: single nucleotide variations
TB: tuberculosis
XDR: extensively-drug resistant
