# Supplementary figures and images for: Whole Genome Sequencing of Mycobacterium tuberculosis Clinical Isolates From India Reveals Genetic Heterogeneity and Region-Specific Variations That Might Affect Drug Susceptibility
Source: Front Microbiol. 2019 Feb 26;10:309. doi: 10.3389/fmicb.2019.00309 (PMC6399466; doi:10.3389/fmicb.2019.00309)

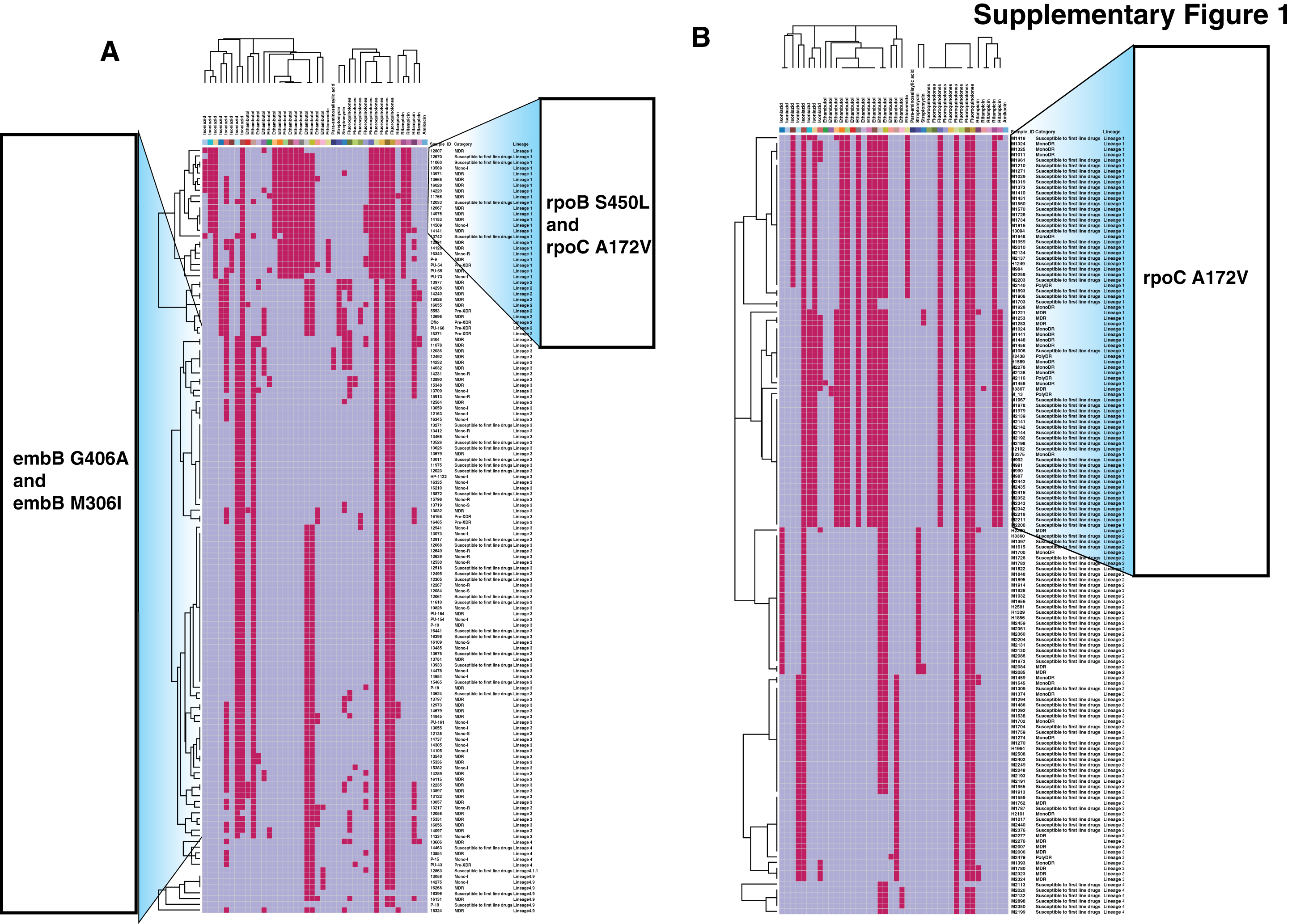

Supplement: FIGURE S1 — Co-occurring mutations in M. tuberculosis clinical isolates. The mutations marked in red are the co-occurring mutations. (A) The highlighted clusters show the prevalence of these mutations in different lineages of M. tuberculosis in isolates from North India. (B) The highlighted clusters show the prevalence of these mutations in different lineages of M. tuberculosis in isolates from South India. [file Image_1.TIF]
